# Longer genotypically-estimated leukocyte telomere length is associated with increased adult glioma risk

**DOI:** 10.18632/oncotarget.6468

**Published:** 2015-12-04

**Authors:** Kyle M. Walsh, Veryan Codd, Terri Rice, Christopher P. Nelson, Ivan V. Smirnov, Lucie S. McCoy, Helen M. Hansen, Edward Elhauge, Juhi Ojha, Stephen S. Francis, Nils R. Madsen, Paige M. Bracci, Alexander R. Pico, Annette M. Molinaro, Tarik Tihan, Mitchel S. Berger, Susan M. Chang, Michael D. Prados, Robert B. Jenkins, Joseph L. Wiemels, ENGAGE Consortium Telomere Group, Nilesh J. Samani, John K. Wiencke, Margaret R. Wrensch

**Affiliations:** ^1^ Division of Neuroepidemiology, Department of Neurological Surgery, University of California, San Francisco, San Francisco, California, USA; ^2^ Program in Neurologic Oncology, Helen Diller Family Comprehensive Cancer Center, University of California, San Francisco, San Francisco, California, USA; ^3^ Department of Cardiovascular Sciences, University of Leicester, Leicester, UK; ^4^ National Institute for Health Research Leicester Cardiovascular Biomedical Research Unit, Glenfield Hospital, Leicester, UK; ^5^ Department of Neurological Surgery, University of California, San Francisco, San Francisco, California, USA; ^6^ Department of Epidemiology and Biostatistics, University of California, San Francisco, San Francisco, California, USA; ^7^ Gladstone Institutes, San Francisco, California, USA; ^8^ Department of Pathology, University of California, San Francisco, San Francisco, California, USA; ^9^ Department of Laboratory Medicine and Pathology, Mayo Clinic College of Medicine, Rochester, Minnesota, USA; ^10^ Institute for Human Genetics, University of California, San Francisco, San Francisco, California, USA; ^11^ Full lists of members and affiliations appear in the Supplementary Note

**Keywords:** single nucleotide polymorphism, telomerase, telomere, CST complex, glioma

## Abstract

Telomere maintenance has emerged as an important molecular feature with impacts on adult glioma susceptibility and prognosis. Whether longer or shorter leukocyte telomere length (LTL) is associated with glioma risk remains elusive and is often confounded by the effects of age and patient treatment. We sought to determine if genotypically-estimated LTL is associated with glioma risk and if inherited single nucleotide polymorphisms (SNPs) that are associated with LTL are glioma risk factors. Using a Mendelian randomization approach, we assessed differences in genotypically-estimated relative LTL in two independent glioma case-control datasets from the UCSF Adult Glioma Study (652 patients and 3735 controls) and The Cancer Genome Atlas (478 non-overlapping patients and 2559 controls). LTL estimates were based on a weighted linear combination of subject genotype at eight SNPs, previously associated with LTL in the ENGAGE Consortium Telomere Project. Mean estimated LTL was 31bp (5.7%) longer in glioma patients than controls in discovery analyses (*P* = 7.82×10-8) and 27bp (5.0%) longer in glioma patients than controls in replication analyses (1.48×10-3). Glioma risk increased monotonically with each increasing septile of LTL (O.R.=1.12; *P* = 3.83×10-12). Four LTL-associated SNPs were significantly associated with glioma risk in pooled analyses, including those in the telomerase component genes TERC (O.R.=1.14; 95% C.I.=1.03-1.28) and TERT (O.R.=1.39; 95% C.I.=1.27-1.52), and those in the CST complex genes OBFC1 (O.R.=1.18; 95% C.I.=1.05-1.33) and CTC1 (O.R.=1.14; 95% C.I.=1.02-1.28). Future work is needed to characterize the role of the CST complex in gliomagenesis and further elucidate the complex balance between ageing, telomere length, and molecular carcinogenesis.

## INTRODUCTION

Gliomagenesis is a complex process, influenced by both inherited and acquired genetic and epigenetic variation. In previous genome-wide association studies (GWAS), we and others have shown that inherited single nucleotide polymorphisms (SNPs) in the telomere-related genes *TERC*, *TERT* and *RTEL1* are associated with increased glioma risk [1-3], suggesting that telomere biology may have a role in gliomagenesis [4]. We recently showed that glioma risk alleles near *TERC* and *TERT* are associated with increased leukocyte telomere length (LTL) [3]. Here we examine the broader hypothesis of whether inherited genetic variation previously associated with LTL is also associated with glioma risk.

Human telomeres, composed of a ­tandem hexanucleotide repeat (TTAGGG), are many kilobases long in the leukocytes of newborns but shorten an average of 20-40 base-pairs annually [5-7]. Because telomere length is highly correlated across tissues [8, 9], LTL is an accessible and increasingly useful marker of human telomere length. In addition to age, inherited genetic variation is a strong determinant of LTL. To-date, eight SNPs near *ACYP2, TERC, NAF1, TERT, OBFC1, CTC1, ZNF208,* and *RTEL1* have been consistently associated with mean LTL in very large GWAS analyses [4, 10].

Recent studies that directly measured LTL in blood specimens from glioma patients and controls have observed conflicting results, possibly due to differences in statistical power or different distributions of potential confounders (*e.g.* age and chemotherapy) [11, 12]. To overcome these limitations, we estimated LTL in two independent datasets of glioma patients and controls based on their genotypes at eight SNPs that have been definitively associated with LTL in a GWAS of 37,684 individuals of European ancestry. Because these estimates of LTL are based on genotypes present since birth, case-control comparisons are not confounded by the effect that age or other factors may have on both telomere length and glioma risk. This study design eliminates the possibility of reverse causation from any effects that the tumor microenvironment or genotoxic therapies may have on telomere attrition. This Mendelian randomization approach for examining the association of genotypically-estimated LTL has been previously applied to other cancers, but not to glioma [13, 14]. We also examined the effect of individual LTL-associated SNPs on glioma risk in a pooled analysis of the full case-control dataset.

## RESULTS

We estimated LTL in glioma cases and controls by creating a weighted linear combination of LTL-associated SNPs. We summed the number of “long LTL” alleles that an individual possesses and weighted each allele by its effect size in data from the ENGAGE Consortium Telomere Group. After excluding samples with imputed genotype probabilities < 0.80 for one or more of the eight LTL-associated SNPs, a total of 652 glioma patients and 3735 controls remained in the discovery dataset and 478 glioma patients and 2559 controls in the replication dataset. The genotypically-estimated relative LTL across individuals ranged from a minimum value of 115bp to a maximum value of 1008bp. This 893bp range in genotypically-estimated LTL corresponds to approximately 30 years of age-related telomere attrition (based on an average LTL attrition rate of 20-40 bp/year). Because the LTL estimates were determined using unlinked autosomal SNPs present since birth, there was no association between genotypically-estimated relative LTL and either subject age or sex (Supplementary Figure 1).

**Figure 1:**
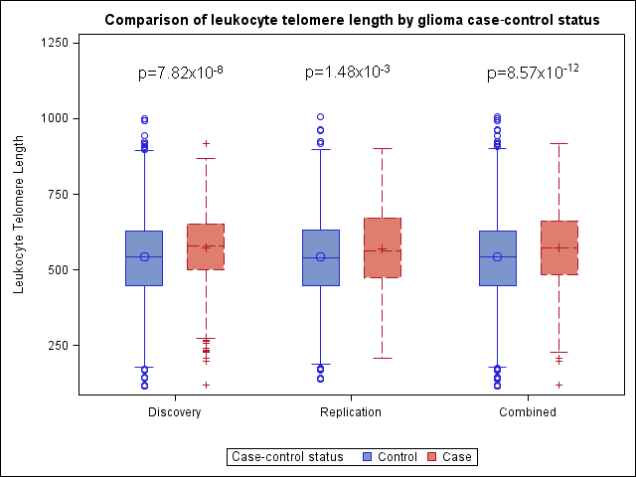
Boxplots comparing genotypically-estimated leukocyte telomere length in glioma patients and controls from discovery analyses (652 glioma patients, 3735 controls), replication analyses (478 glioma patients, 2559 controls), and combined analyses (1130 glioma patients, 6294 controls); glioma cases from the UCSF Adult Glioma Study (AGS) and TCGA, and controls from AGS, Illumina iControls and Wellcome-Trust *P*-values are adjusted for the first two ancestry-informative principal components and, in the combined analysis, for genotyping platform.

In the discovery dataset, the average genotypically-estimated relative LTL value was significantly longer in glioma cases (573bp) than in controls (542bp) (*P* = 7.82×10^−8^) (Figure 1). Similar results were observed in the replication dataset, with the average genotypically-estimated relative LTL again being significantly longer in glioma cases (571bp) than in controls (544bp) (1.48×10^−3^) (Figure 1), providing strong support for both the direction and magnitude of the association. LTL estimates were similar for both glioblastoma patients (569bp) and lower-grade glioma patients (574bp) from the TCGA replication set, suggesting no differences in the effect of LTL across strata of WHO tumor grade (Grade IV versus Grade II/III). LTL estimates were also similar in all three control groups (AGS = 547bp, iControl = 541bp, WTCCC = 544bp; *P* = 0.86).

**Figure 2:**
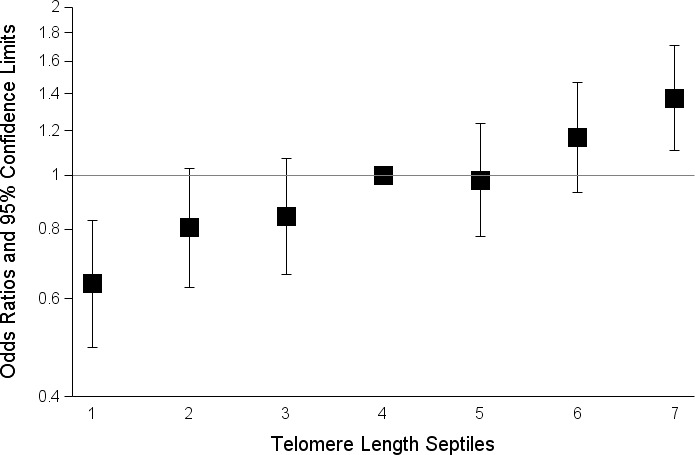
Effect of increasing septile of genotypically-estimated leukocyte telomere length on glioma risk in combined discovery and replication datasets The odds ratios are relative to the median (fourth) septile. Vertical bars correspond to 95% confidence intervals. Septiles were defined among controls and ranges were: septile one (115bp-403bp), septile two (404bp-467bp), septile three (468bp-523bp), septile four (524bp-567bp), septile five (568bp-616bp), septile six (617bp-684bp), septile seven (685bp-1008bp).

**Figure 3:**
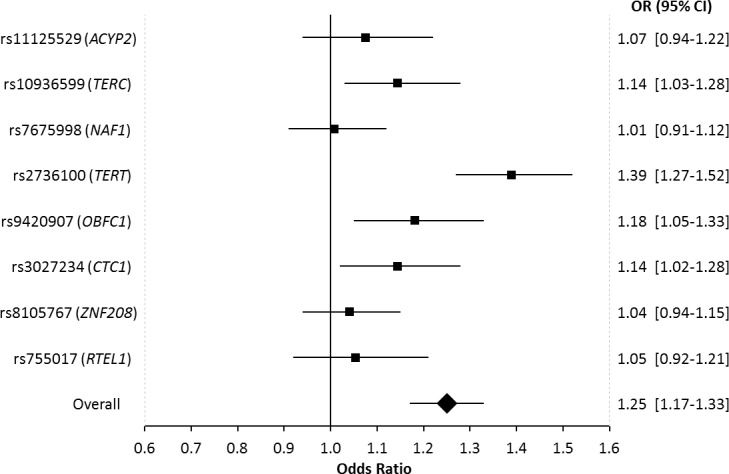
Forest plot showing the effect of alleles associated with longer leukocyte telomere length on glioma risk Allelic odds ratios are plotted with 95% confidence intervals. The overall estimate is for the combined effect of all 8 SNPs, where the odds ratio relates to the change in glioma risk for one standard deviation increase in genotypically-estimated leukocyte telomere length. Odds ratios are based on combined data from 1130 glioma patients and 6294 controls.

In pooled analyses of the combined discovery and replication datasets, glioma patients had genotypically-estimated LTL that was, on average, ~5.3% longer than that of controls (572bp vs. 543bp; *P* = 8.57×10^−12^) (Figure 1). Moreover, the odds ratio for glioma increased monotonically with each increasing septile of LTL (O.R. = 1.12; 95%C.I. = 1.09-1.16; *P* = 3.83×10^−12^) (Figure 2). Individuals in the highest LTL septile had a 2.2-fold increased risk of glioma compared to individuals in the lowest LTL septile (O.R. = 2.16; 95% C.I. = 1.68-2.76; *P* = 1.45×10^−9^). A one standard deviation increase in genotypically-estimated LTL was associated with a 1.25-fold increased risk of glioma (OR = 1.25; 95%C.I. = 1.17-1.33; *P* = 1.19×10^−11^) (Figure 3).

To determine whether the association of glioma risk with genotypically-estimated LTL was due only to the previously established associations of glioma risk with SNPs near *TERC*, *TERT* and *RTEL1*, LTL estimates were re-calculated in the combined discovery and replication dataset using the other five LTL-associated SNPs located near genes that had not been previously associated with glioma risk (*ACYP2*, *NAF1*, *OBFC1*, *CTC1* and *ZNF208*). Using this reduced 5-SNP estimate of genotypically-estimated relative LTL, glioma patients still had 2.8% longer estimated LTL than controls (259bp vs. 252bp; *P* = 0.011) (Supplementary Figure 2). This suggests that additional LTL-associated SNPs outside the *TERC*, *TERT* and *RTEL1* regions also confer glioma risk.

In single-locus analyses of each of the eight LTL-associated SNPs, four of the eight SNPs were associated with glioma risk at *P* < 0.05, including: rs10936599 in *TERC* (O.R. = 1.14; 95% C.I. = 1.03-1.28; *P* = 0.014) and rs2736100 in *TERT* (O.R. = 1.39; 95%C.I. = 1.27-1.52; *P* = 9.5×10^−13^) (as previously reported) and novel associations with glioma risk were observed at rs9420907 in *OBFC1* (O.R. = 1.18; 95%C.I. = 1.05-1.33; *P* = 7.3×10^−3^) and rs3027234 in *CTC1* (O.R. = 1.14; 95%C.I. = 1.02-1.28; *P* = 0.020) (Table 1, Figure 3). For all eight LTL-associated SNPs, the allele that was previously associated with longer LTL was observed more commonly in cases than in controls (*P*_sign-test_ = 7.8×10^−3^) (Supplementary Figure 3A). Furthermore, the proportion of variance in LTL explained by each SNP was positively but non-significantly correlated with the proportion of variance in glioma risk explained by that SNP (*r* = 0.55, *P* = 0.16) (Supplementary Figure 3B).

**Table 1:** Results for each telomere length-associated SNP, including effect on telomere length in the ENGAGE Consortium genome-wide meta-analysis and on glioma risk in the combined UCSF Adult Glioma Study (AGS), The Cancer Genome Atlas (TCGA), and Wellcome Trust Case-Control Consortium (WTCCC) data

SNP^a^	Chromosome	Gene	Effect Allele^b^	Effect on LTL(ENGAGE Consortium)				Effect on glioma risk(UCSF AGS, TCGA, WTCCC)			
				EAF^c^	Beta	BP^d^	P	EAF^e^	OR^f^	95% CI	P
rs11125529	2	ACYP2	A	14%	0.056	66.9	4.5×10^−8^	13%	1.07	0.94-1.22	0.28
rs10936599	3	TERC	C	75%	0.097	117.3	2.5×10^−31^	76%	1.14	1.03-1.28	0.014
rs7675998	4	NAF1	G	78%	0.074	89.7	4.3×10^−16^	77%	1.01	0.91-1.12	0.89
rs2736100	5	TERT	C	49%	0.078	94.2	4.4×10^−19^	51%	1.39	1.27-1.52	9.5×10^−13^
rs9420907	10	OBFC1	C	14%	0.069	82.8	6.9×10^−11^	14%	1.18	1.05-1.33	7.3×10^−3^
rs3027234	17	CTC1	C	79%	0.021	25.2	0.020	78%	1.14	1.02-1.28	0.020
rs8105767	19	ZNF208	G	29%	0.048	57.6	1.1×10^−9^	29%	1.04	0.94-1.15	0.43
rs755017	20	RTEL1	G	13%	0.062	74.1	6.7×10^−9^	12%	1.05	0.92-1.21	0.46

a All SNPs were associated with LTL in a previous GWAS and replicated at P < 0.05 in the ENGAGE Consortium genomewide meta-analysis [4]

b The effect allele is the allele associated with increased leukocyte telomere length.

c Effect allele frequency (EAF) calculated in all ENGAGE Consortium subjects (N=37,684)

d Base pair (BP) estimates of the per-allele effect on LTL in base pairs calculated from the equivalent age-related attrition in telomere repeat length ratio, as previously described [4]

e Effect allele frequency (EAF) calculated in glioma control subjects

f Odds ratios (OR) are for each additional copy of the allele associated with longer LTL. Odds ratios >1.0 indicate that the “long” allele is more common in glioma patients and the “short” allele is more common in controls.

## DISCUSSION

Observational data support a connection between both longer and shorter telomere length and increased cancer risk [15-17]. Meta-analyses indicate that the direction of association may be tumor-specific [16] and that there may be differences in the importance of shorter or longer telomere length at different stages of carcinogenesis [18]. Short or unprotected telomeres can form telomeric fusions, leading to genomic instability - a hallmark of cancer [19]. Conversely, telomere depletion ultimately induces replicative senescence and limits the proliferative capacity of cells. Therefore, a predisposition to long telomeres may permit cells to escape growth arrest and undergo malignant transformation. Our results indicate that a genetic predisposition to *longer* LTL is associated with *increased* glioma risk. This is not unprecedented, as recent reports using similar Mendelian randomization approaches have observed strong association between longer genotypically-estimated LTL and increased risk of melanoma and lung adenocarcinoma, but no association was observed for breast, colorectal, ovarian or prostate cancer [13, 14].

Previous studies that directly measured LTL in glioma patients and controls have observed mixed results [11, 12]. The first of these studies, which included 101 glioma patients and 198 healthy controls, identified a non-significant association between longer LTL and increased glioma risk in female subjects and the inverse in male subjects. A larger study measuring mean LTL in 467 adult glioma patients and 467 age and sex-matched controls observed a significant non-linear relationship between LTL and glioma risk [11]. Specifically, individuals in both the upper and lower tertile of LTL had increased risk of glioma relative to individuals in the middle tertile. Our data revealed a consistent dose-response relationship between glioma risk and septiles of genotypically-estimated LTL. Because our large sample size permitted finer stratification of LTL exposure groups (*i.e.* septiles versus tertiles), we can be more confident in the linearity of the relationship we observe. Furthermore, our estimates of LTL rely solely on inherited genetic variation, present since birth, and the association is therefore less prone to the confounding or biasing effects of age, sex, patient treatment, or other factors.

Mendelian randomization is an epidemiologic technique in which genetic variants that are known to influence an exposure of interest (*e.g.* LTL) are used as surrogate biomarkers to investigate the effect of that exposure on a disease of interest (*e.g.* glioma) [20]. Although each of the LTL-associated variants explains only a small proportion of the total variance in telomere length across individuals [4], a summary variable made by combining the eight SNPs accounted for an 893bp difference in genotypically-estimated LTL - corresponding to a nearly 30 years of age-related telomere attrition [21]. Because genotypes are randomized at birth, Mendelian randomization studies can inform on the causality of associations by controlling for both confounding and reverse causation.

Although Mendelian randomization designs reduce confounding and bias, study results can be influenced by linkage disequilibrium, population stratification, and pleiotropy [22]. Because we analyzed unlinked SNPs on separate autosomes, linkage disequilibrium is unlikely to influence our results. By carefully excluding individuals with non-European ancestry and adjusting for principal components in all analyses, inflation of test statistics due to population stratification is unlikely. There remains a possibility that pleiotropy could underlie the association between LTL and glioma risk observed in our data. Genetic variation near *TERT* and *TERC* may influence cancer risk independent of its effect on telomere length, as telomerase has been shown to upregulate glycolysis and may contribute to the Warburg effect [23]. In addition to maintaining telomere stability, the RTEL1 protein stabilizes DNA replication forks which could also influence tumorigenesis [24]. Because the association between glioma risk and LTL remained significant even after excluding the *TERC*, *TERT* and *RTEL1* SNPs from the LTL calculations, it is unlikely that the association observed in our data could be entirely attributable to pleiotropy. However, additional functions of the *ACYP2*, *NAF1*, *OBFC1*, *CTC1* and *ZNF208* loci are not precluded by our observations.

The allele associated with longer LTL was associated with increased glioma risk at each of the eight LTL-associated SNPs. However, it remains possible that longer telomere length is not itself a causal factor in gliomagenesis, but rather may be a biomarker of cells that are more susceptible to telomerase reactivation or some related factor. Although the proportion of variance in LTL explained by each SNP was positively correlated with the proportion of variance in glioma risk explained by each SNP, this association was not statistically significant and suggests that tissue-specific differences in the regulation of telomere length may be an important consideration in future studies.

A growing body of epidemiologic and tumor genomic research has identified an important role for telomerase in glioma predisposition, initiation and prognosis [1, 25, 26]. Somatic mutations in the promoter of *TERT* are found in a large proportion of adult gliomas [26, 27], where they lead to aberrant binding of the GABP transcription factor and telomerase reactivation [28]. Our single-locus analyses show an association between inherited variation in the telomerase component genes *TERC* and *TERT* and glioma risk, as previously reported [1-3, 29].

Although we previously described a link between the telomerase component genes (*TERC* and *TERT*) and glioma risk, this is the first report of a significant association between CST complex genes (*CTC1* and *OBFC1*) and glioma risk. The human CST complex is encoded by three genes: *CTC1*, *OBFC1* and *TEN1*, and it competes with shelterin for telomeric DNA and inhibits telomerase-based telomere extension. Through binding of the telomerase-extended telomere, CST limits telomerase activity and restricts telomere extension to approximately one event per cell-cycle [30]. The significant association between glioma risk and common LTL-associated variants in *CTC1* and *OBFC1* is particularly intriguing in light of a recent report demonstrating that germline loss-of-function mutations in shelterin-complex genes are a rare cause of familial oligodendroglioma [31]. Future work should identify the full suite of genetic variants involved in telomere maintenance and characterize their relationship to glioma risk. Such studies will likely benefit from the incorporation of both inherited (constitutive) and acquired (tumor) variants into a comprehensive model of gliomagenesis [32].

## MATERIALS AND METHODS

### Ethics statement

Glioma studies were approved by the University of California, San Francisco Committee on Human Research. Informed consent was obtained from all study participants. The genome-wide meta-analysis of mean leukocyte telomere length (LTL) obtained approval by local ethics committees, as previously outlined [4].

### Estimating relative LTL based on inherited genotype in LTL-associated SNPs

A total of 37,684 individuals of European descent, aged >18 years, had LTL measurements available and were used to determine the effect size of the 8 SNPs associated with LTL in prior GWAS. These individuals were from 15 cohorts collected and analyzed by the ENGAGE Consortium Telomere Group, comprised of European and Australian collaborating institutions [4]. All cohorts for the LTL genome-wide meta-analysis had genotype information generated on a standard genotyping platform from Illumina or Affymetrix, and include imputed genotypes based on HapMapII CEU reference data, as previously published [4]. SNPs from the eight regions most strongly associated with LTL in this and other studies include: rs11125529 (*ACYP2*), rs10936599 (*TERC*), rs7675998 (*NAF1*), rs2736100 (*TERT*), rs9420907 (*OBFC1*), rs3027234 (*CTC1*), rs8105767 (*ZNF208*), and rs755017 (*RTEL1*). At each of these SNPs, one allele is associated with longer LTL and the alternate allele is associated with shorter LTL. Thus, each individual has from 0 to 16 alleles associated with longer LTL.

We estimated LTL in glioma cases and controls by creating a weighted linear combination of LTL-associated SNPs. We summed the number of “long LTL” alleles that an individual possesses and weighted each allele by its effect size in data from the ENGAGE Consortium Telomere Group (effect sizes appear in Table 1) [4]. The effect size used for weighting was expressed as the number of additional base-pairs of telomere length associated with each allele, adjusted for age and sex, as calculated by the ENGAGE Consortium Telomere Group [4]. The number of base-pairs was used because the output of the model can be interpreted as the relative difference in estimated LTL across individuals. The model assigns a value of “0” to an individual who possesses 0 of the alleles associated with longer LTL, while an individual possessing all sixteen alleles would have a value of “1215”. This can be interpreted as a 1215 base-pair (bp) difference in genotypically-estimated mean LTL between two such individuals. The effect size of each allele on LTL within the ENGAGE consortium data appears in Table 1 [4].

### LTL population genotyping

All cohorts for the genome-wide meta-analysis of leukocyte telomere length had genotype information generated on a standard genotyping platform from Illumina or Affymetrix, and include imputed genotypes based on HapMapII CEU reference data, as previously published [4].

### Glioma case-control study groups

We calculated genotypically-estimated LTL among individuals in two independent glioma case-control study groups (Supplementary Table 1). All individuals were of European ancestry and cases were older than 18 years of age. The discovery group included 620 UCSF Adult Glioma Study (AGS) high-grade glioma patients (85% glioblastoma, 15% grade III astrocytoma), 70 TCGA glioblastoma patients, 602 AGS control subjects, and 3390 Illumina iControl subjects genotyped on Illumina SNP arrays [1].

AGS cases were newly diagnosed patients with histologically confirmed high-grade glioma. Population-based cases from six San Francisco Bay Area counties were ascertained using the Cancer Prevention Institute of California's early case ascertainment system from May 1997 to August 1999 (Series 2), and from November 2001 to September 2005 (Series 3). Clinic-based cases diagnosed between 2002-2006 (Series 3) of the same histologies were recruited from the UCSF Neuro-oncology Clinic, regardless of place of residence. From 1991-2010, population-based controls from the same residential area as the population-based cases were identified using random digit dialing and were frequency matched to population-based cases on age, gender and ethnicity. Tumor specimens and pathology reports were reviewed by UCSF neuropathologists. Consenting participants provided blood, buccal and/or saliva specimens and information during an in-person or telephone interview.

Replication analyses included 499 non-overlapping glioma patients from TCGA (65% glioblastoma, 35% grades II-III glioma) and 2603 controls from the WTCCC, genotyped on Affymetrix SNP arrays [33, 34]. All individuals were of European ancestry.

### Glioma case-control genotyping and imputation

For AGS cases and controls, DNA was isolated either from fresh whole blood using an automated extraction system (Autogen, Inc) or from frozen whole blood aliquots using the Gentra Puregene DNA isolation kit (Qiagen). Sample concentration was quantified using Picogreen reagent (Invitrogen). All samples in the discovery analysis were genotyped using either the Illumina HumanCNV370-Duo BeadChip or the Illumina HumanHap550 platform, as previously described [1]. Briefly, a total of 51 duplicate samples were plated with average concordance >99%. Samples with call rates < 98% were excluded from analysis, as were samples with mismatched reported and genotyped sex. Although all subjects were of self-reported European-ancestry, this was validated using principal components analysis in Eigenstrat [35]. Analyzed SNPs had call rates >98% and Hardy-Weinberg equilibrium p-values >0.001 among controls.

All samples in the replication analysis were genotyped using the Affymetrix 6.0 genotyping array. Genotyping data for 499 glioma patients, not included in discovery analyses, were downloaded from TCGA [34]. Genotype data for 2603 European-ancestry control samples were downloaded from the Wellcome Trust Case-Control Consortium [33]. Subjects showing evidence of non-European ancestry, as well as duplicate samples and related subjects (IBS > 1.6) among the TCGA cases and WTCCC controls were excluded from analyses. Genome-wide SNP data were used to ensure there was no overlap between TCGA glioma patients and AGS glioma patients. SNPs with call rates < 98% or HWE *p*-value < 0.001 among controls were excluded.

Within the two glioma case-control datasets, we imputed 100kb regions centered on eight SNPs previously associated with LTL in GWAS [4, 7, 10, 36]: rs11125529 (*ACYP2*), rs10936599 (*TERC*), rs7675998 (*NAF1*), rs2736100 (*TERT*), rs9420907 (*OBFC1*), rs3027234 (*CTC1*), rs8105767 (*ZNF208*), and rs755017 (*RTEL1*). In the discovery dataset, the top LTL SNP was directly genotyped on an Illumina array for *TERC*, *TERT* and *OBFC1* and was imputed for the other five genes. In the replication dataset, the top LTL SNP was directly genotyped on an Affymetrix array for *ACYP2*, *TERT*, *OBFC1*, *CTC1* and *ZNF208* and was imputed for the other three genes. Imputation was performed using the Impute2 v2.1.2 software and its standard Markov chain Monte Carlo algorithm and default settings for targeted imputation [37]. All 1,000 Genomes Phase I haplotypes were provided as the imputation reference panel [38]. All SNPs had imputation quality (info) scores > 0.80 and posterior probabilities > 0.90. Individuals with imputed genotype probabilities < 0.80 were excluded from analyses to prevent allele misclassification and minimize the effect of poor SNP imputation on LTL estimation. Imputation was performed separately for the discovery dataset (Illumina array data) and the replication dataset (Affymetrix data).

### Statistical analyses

Differences in genotypically-estimated LTL were compared between glioma patients and controls in the discovery analysis using logistic regression, with adjustment for the first 2 ancestry-informative principal components from Eigenstrat [35]. The same method was applied to the replication set. Summary results for all patients and controls were generated in pooled logistic regression analyses, adjusting for principal components and array type (Illumina 370k vs. Affymetrix 6.0). Additionally, septiles of LTL were determined among the pooled control set and applied to glioma patients to calculate odds ratios associated with increasing septiles of LTL. Additionally, we calculated odds ratios corresponding to the change in glioma risk relative to a one standard deviation increase in genotypically-estimated LTL (with the standard deviation defined among the pooled controls).

For single locus SNP associations in the pooled dataset, statistics for imputed and directly genotyped SNPs were calculated using logistic regression in SNPTESTv2 using an allelic additive model [39], adjusting for the first 2 principal components from Eigenstrat [35] and array type. To account for potential errors in imputation, a missing-data likelihood score-test was applied to the imputed variants to produce standard errors which account for the additional uncertainty inherent in the analysis of imputed genotypes. The proportion of variance in glioma risk explained by a single SNP was determined using the Cox-Snell R^2^ statistic from logistic regression analyses. The proportion of variance in LTL explained by a single SNP was determined using the standard Pearson R^2^ statistic from linear regression analyses.

At each of the eight SNPs, either the allele associated with longer telomeres or the allele associated with shorter telomeres will have a higher frequency in cases than in controls. We sought to determine if the “long” allele or the “short” was more frequent in cases than would be expected under the null hypothesis (no association between LTL and glioma risk). To do so we applied a sign-test based on observations at each of the eight SNPs, with probabilities calculated using the binomial test (Probability that the “long” allele is more common in cases than controls = Probability that the “short” allele is more common in cases than controls = 0.50).

## SUPPLEMENTARY MATERIAL FIGURES AND TABLE


