# Supplementary material for: Longer genotypically-estimated leukocyte telomere length is associated with increased adult glioma risk
Source: Oncotarget. 2015 Dec 4;6(40):42468–77. doi: 10.18632/oncotarget.6468 (PMC4767445; doi:10.18632/oncotarget.6468)
Supplement: Supplementary file 1 [file oncotarget-06-42468-s001.pdf]

Longer genotypically-estimated leukocyte telomere length is associated with increased adult glioma risk

Supplementary Material

SUPPLEMENTARY NOTE: ENGAGE Consortium Telomere Group members and affiliations

European Network for Genetic and Genomic Epidemiology (ENGAGE) Consortium Telomere Group members and affiliations

Veryan Codd[1,2], Christopher P. Nelson[1,2], Eva Albrecht[3], Massimo Mangino[4], Joris Deelen[5,6], Jessica L. Buxton[7], Jouke Jan Hottenga[8], Krista Fischer[9], Tõnu Esko[9], Ida Surakka[10,11], Linda Broer[6,12,13], Dale R. Nyholt[14],Irene Mateo Leach[15], Perttu Salo[11], Sara Hägg[16], Mary K. Matthews[1], Jutta Palmen[17], Giuseppe D. Norata[18-20], Paul F. O'Reilly[21,22], Danish Saleheen[23,24], Najaf Amin[12], Anthony J. Balmforth[25],Marian Beekman[5,6], Rudolf A. de Boer[15],Stefan Böhringer[26], Peter S. Braund[1], Paul R. Burton[27],Anton J. M. de Craen[28], Matthew Denniff[1], Yanbin Dong[29], Konstantinos Douroudis[9], Elena Dubinina[1], Johan G. Eriksson[11,30-32], Katia Garlaschelli[19], Dehuang Guo[29], Anna-Liisa Hartikainen[33], Anjali K. Henders[14], Jeanine J. Houwing-Duistermaat[6,26], Laura Kananen[34,35], Lennart C. Karssen[12], Johannes Kettunen[10,11], Norman Klopp[36,37], Vasiliki Lagou[38], Elisabeth M. van Leeuwen[12], Pamela A. Madden[39], Reedik Mägi[9], Patrik K.E. Magnusson[16], Satu Männistö[11], Mark I. McCarthy[38,40,41], Sarah E. Medland[14], Evelin Mihailov[9], Grant W. Montgomery[14], Ben A. Oostra[12], Aarno Palotie[42,43], Annette Peters[36,44,45], Helen Pollard[1], Anneli Pouta[33,46], Inga Prokopenko[38], Samuli Ripatti[10,11,42], Veikko Salomaa[11], H. Eka D. Suchiman[5], Ana M.Valdes[4], Niek Verweij[15], Ana Viñuela[4], Xiaoling Wang[29], H.-Erich Wichmann[47,48,49], Elisabeth Widen[10], Gonneke Willemsen[8], Margaret J. Wright[14], Kai Xia[50], Xiangjun Xiao[51],Dirk J. van Veldhuisen[15], Alberico L. Catapano[18,52], Martin D. Tobin[27], Alistair S. Hall[25], Alexandra I.F. Blakemore[7], Wiek H. van Gilst[15], Haidong Zhu[29], Jeanette Erdmann[53], Muredach P. Reilly[54], Sekar Kathiresan[55-57], Heribert Schunkert[53], Philippa J. Talmud[17], Nancy L. Pedersen[16], Markus Perola[9-11,], Willem Ouwehand[42,58,59], Jaakko Kaprio[10,60,61], Nicholas G. Martin[14], Cornelia M. van Duijn[6,12,13], Iiris Hovatta[34,35,61], Christian Gieger[3], Andres Metspalu[9], Dorret I. Boomsma[8], Marjo-Riitta Jarvelin[21,22,62-64], P. Eline Slagboom[5,6], John R. Thompson[27], Tim D. Spector[4], Pim van der Harst[1,15,65], Nilesh J. Samani[1,2].

[1]Department of Cardiovascular Sciences, University of Leicester, Leicester, UK
[2]NIHR Leicester Cardiovascular Biomedical Research Unit, Glenfield Hospital, Leicester, UK
[3]Institute of Genetic Epidemiology, Helmholtz Zentrum München - German Research Center for Environmental Health, Neuherberg, Germany
[4]Department of Twin Research and Genetic Epidemiology, King's College London, London, UK
[5]Section of Molecular Epidemiology, Leiden University Medical Center, Leiden, The Netherlands
[6]Netherlands Consortium for Healthy Aging, Leiden University Medical Center, Leiden, The Netherlands
[7]Section of Investigative Medicine, Imperial College London, London, UK
[8]Netherlands Twin Register, Department of Biological Psychology, VU University, Amsterdam, The Netherlands
[9]Estonian Genome Center, University of Tartu, Tartu, Estonia
[10]Institute for Molecular Medicine Finland (FIMM), University of Helsinki, Helsinki, Finland
[11]Public Health Genomics Unit, Department of Chronic Disease Prevention, National Institute for Health and Welfare, Helsinki, Finland
[12]Department of Epidemiology, Erasmus Medical Center, Rotterdam, the Netherlands
[13]Centre for Medical Systems Biology, Leiden, The Netherlands
[14]Queensland Institute of Medical Research, Brisbane, Australia
[15]Department of Cardiology, University of Groningen, University Medical Center, Groningen, The Netherlands
[16]Department of Medical Epidemiology and Biostatistics, Karolinska Institutet, Stockholm, Sweden

[17]Institute of Cardiovascular Science, Univerisity College London, London , UK
[18]Department of Pharmacological and Biomolecular Sciences, Università degli Studi di Milano, Milan, Italy
[19]Centro SISA per lo Studio dell'Aterosclerosi, Bassini Hospital, Cinisello B, Italy
[20]The Blizard Institute, Barts and the London School of Medicine and Dentistry, Queen Mary University, London, UK
[21]Department of Epidemiology and Biostatistics, School of Public Health, Imperial College, London, UK
[22]MRC-HPA Centre for Environment and Health, Faculty of Medicine, Imperial College London, UK
[23]Department of Public Health and Primary Care, University of Cambridge, Cambridge, UK
[24]Center for Non-Communicable Diseases, Karachi, Pakistan
[25]Division of Epidemiology, LIGHT, School of Medicine, University of Leeds, Leeds, UK
[26]Section of Medical Statistics, Leiden University Medical Center, Leiden, The Netherlands
[27]Department of Health Sciences, University of Leicester, Leicester, UK
[28]Department of Gerontology and Geriatrics, Leiden University Medical Center, Leiden, The Netherlands
[29]Georgia Prevention Institute, Georgia Health Sciences University, Augusta, GA, USA
[30]University of Helsinki, Department of General Practice and Primary Health Care, Helsinki, Finland
[31]Folkhälsan Research Center, Helsinki, Finland
[32]Unit of General Practice, Helsinki University Central Hospital, Helsinki, Finland
[33]Institute of Clinical Medicine/Obstetrics and Gynecology, University of Oulu, Oulu, Finland
[34]Research Programs Unit, Molecular Neurology, Biomedicum Helsinki, University of Helsinki, Finland
[35]Department of Medical Genetics, Haartman Institute, University of Helsinki, Finland
[36]Research Unit of Molecular Epidemiology, Helmholtz Zentrum München - German Research Center for Environmental Health, Neuherberg, Germany
[37]Hanover Unified Biobank, Hanover Medical School, Hanover, Germany
[38]Oxford Centre for Diabetes, Endocrinology and Metabolism, University of Oxford, Oxford, UK
[39]Department of Psychiatry, Washington University School of Medicine, St. Louis, MO, USA
[40]Wellcome Trust Centre for Human Genetics, University of Oxford, Oxford, UK
[41]Oxford NIHR Biomedical Research Centre, Churchill Hospital, Oxford, UK
[42]Wellcome Trust Sanger Institute, Wellcome Trust Genome Campus, Hinxton, UK
[43]Department of Medical Genetics, University of Helsinki and the Helsinki University Hospital, Helsinki, Finland
[44]Institute of Epidemiology II, Helmholtz Zentrum München - German Research Center for Environmental Health, Neuherberg, Germany
[45]Munich Heart Alliance, Munich, Germany
[46]National Institute for Health and Welfare, Oulu, Finland
[47]Institute of Epidemiology I, Helmholtz Zentrum München - German Research Center for Environmental Health, Neuherberg, Germany
[48]Institute of Medical Informatics, Biometry and Epidemiology, Chair of Epidemiology, Ludwig-Maximilians-Universität, Munich, Germany
[49]KlinikumGrosshadern, Munich, Germany
[50]Department of Biostatistics, University of North Carolina, Chapel Hill, NC USA
[51]Department of Epidemiology, University of Texas M.D. Anderson Cancer Center, Houston, TX, USA
[52]IRCCS Multimedica, Milan, Italy
[53]UniversitätzuLübeck, Medizinische Klinik II, Lübeck, Germany
[54]The Cardiovascular Institute, University of Pennsylvania, Philadelphia, Pennsylvania, USA
[55]Cardiovascular Research Center and Cardiology Division, Massachusetts General Hospital, Boston, Massachusetts, USA.
[56]Center for Human Genetic Research, Massachusetts General Hospital, Boston, Massachusetts, USA.
[57]Program in Medical and Population Genetics, Broad Institute of MIT and Harvard, Cambridge, Massachusetts, USA
[58]Department of Haematology, University of Cambridge, Cambridge, UK

[59] National Health Service Blood and Transplant, Cambridge, UK
[60]University of Helsinki, Hjelt Institute, Department of Public Health, Helsinki, Finland
[61] Department of Mental Health and Substance Abuse Services, National Institute for Health and Welfare, Helsinki, Finland
[62]Institute of Health Sciences, University of Oulu, Oulu, Finland
[63] Biocenter Oulu, University of Oulu, Oulu, Finland
[64] Department of Lifecourse and Services, National Institute for Health and Welfare, Oulu, Finland
[65]Department of Genetics, University of Groningen, University Medical Center, Groningen, The Netherland

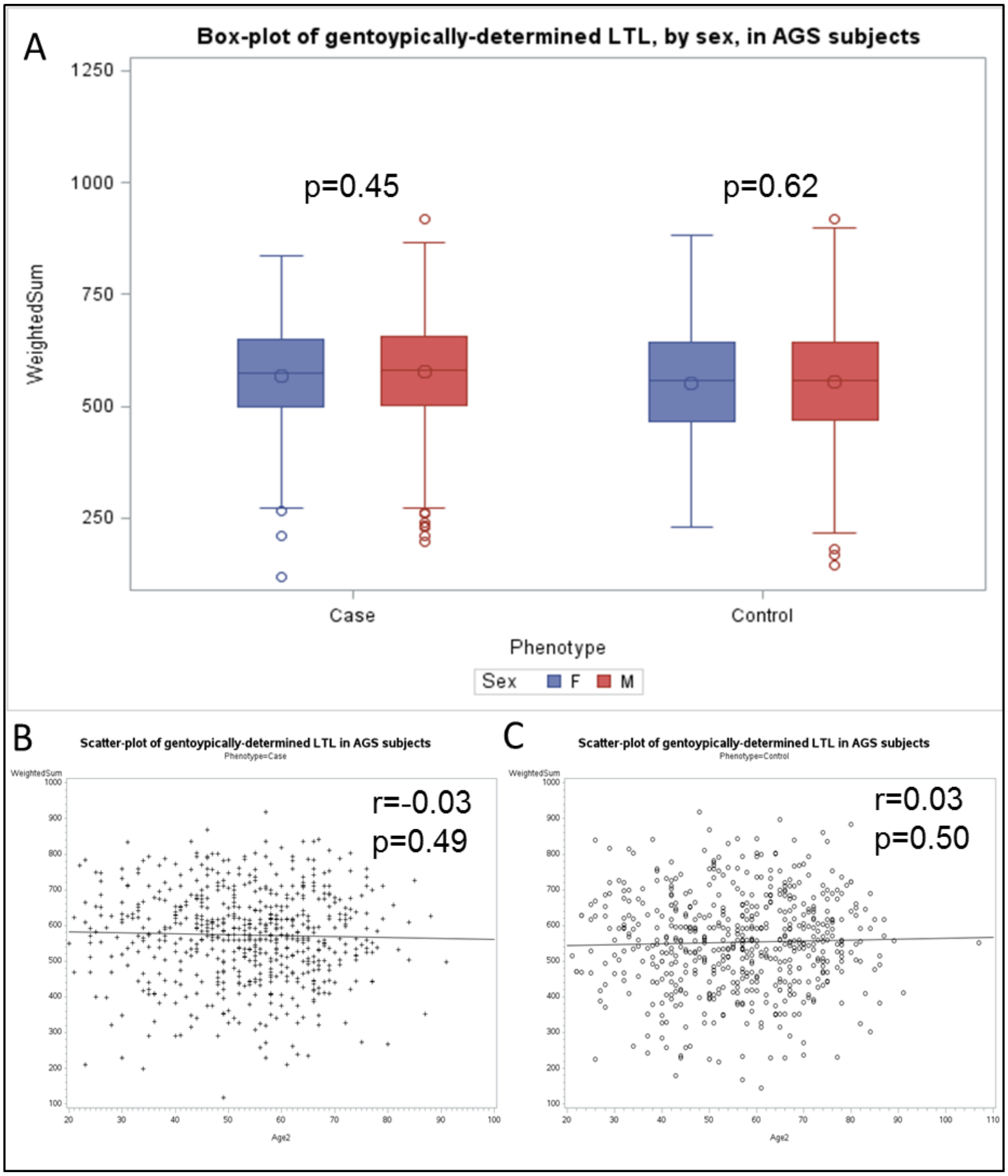

Supplementary Figure 1: Genotypically-estimated leukocyte telomere length (LTL) is not associated with subject age or sex. (A) Boxplots comparing genotypically-estimated LTL in females (blue) and males (red), stratified by glioma case-control status, in AGS subjects. (B) Scatter-plot of genotypically-estimated LTL and subject age in AGS glioma patients. (C) Scatter-plot of genotypically-estimated LTL and subject age in AGS glioma controls.

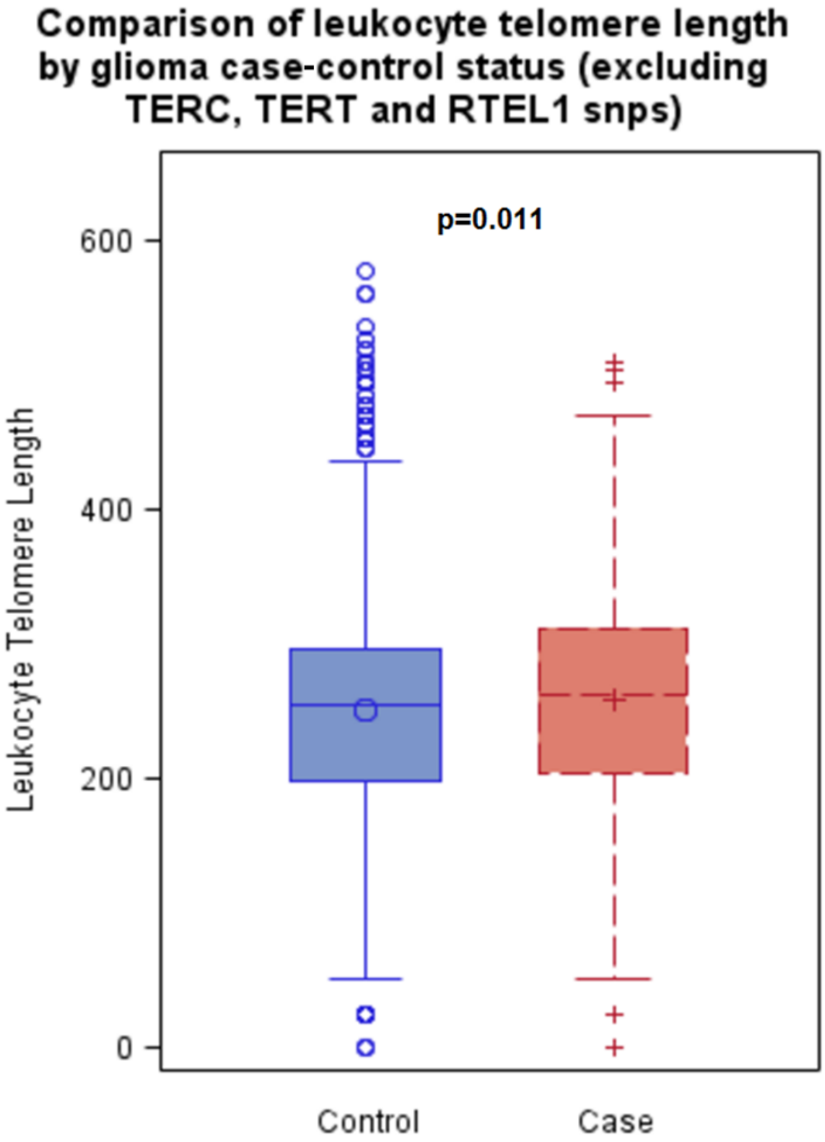

Supplementary Figure 2: Boxplot comparing genotypically-estimated leukocyte telomere length (LTL) in 1130 glioma patients and 6294 controls from the UCSF AGS, TCGA, and WTCCC after excluding contributions from known glioma risk genes (*TERC*, *TERT*, and *RTEL1*) and retaining contributions from *ACYP2*, *NAF1*, *OBFC1*, *CTC1* and *ZNF208*. P-values are adjusted for the first two ancestry-informative principal components and for genotyping platform.

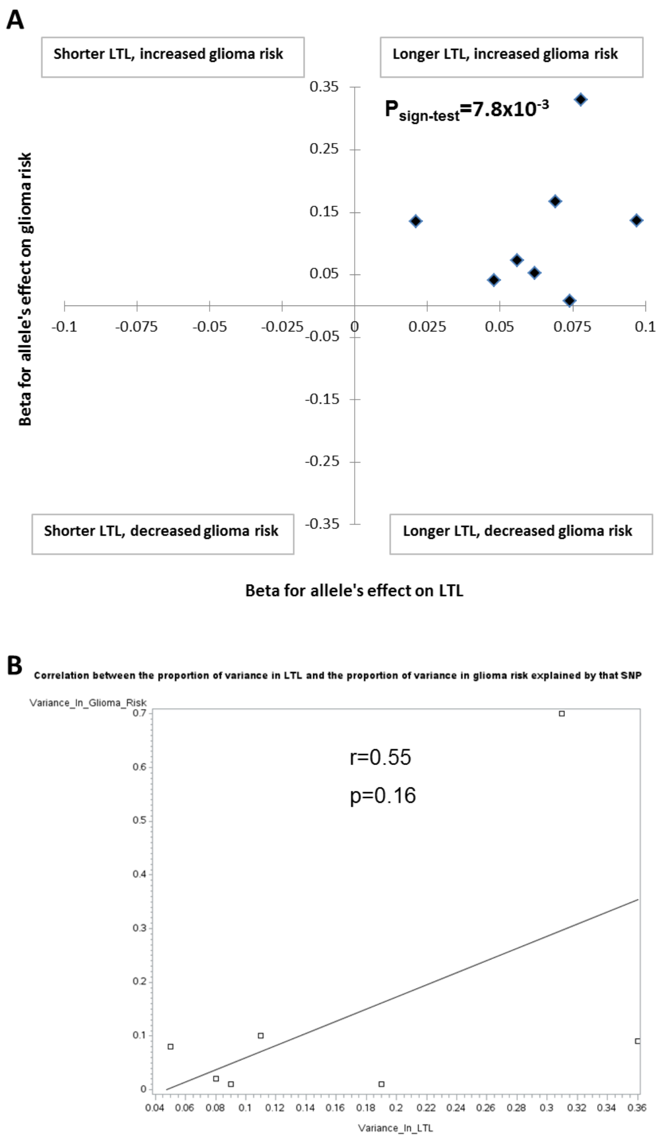

Supplementary Figure 3. Associations between the effects of eight SNPs on leukocyte telomere length (LTL) in the ENGAGE Consortium and glioma risk in the UCSF AGS, TCGA, and WTCCC. (A) For all eight LTL-associated SNPs, the allele associated with longer LTL was associated with increased glioma risk ($P_{sign\text{-}test}=7.8x10^{-3}$). (B) The proportion of variance in LTL explained by each SNP was positively but non-significantly correlated with the proportion of variance in glioma risk explained by that SNP (r=0.55, P=0.16). The proportion of variance explained is expressed as a percentage.

Supplementary Table 1. Subject characteristics of glioma patients and controls, the University of California, San Francisco (UCSF) Bay Area Adult Glioma Study (AGS) 1997-2011, Illumina iControls, The Cancer Genome Atlas (TCGA) glioma patients and Wellcome Trust (WTCCC) controls.

Populations	N (cases/controls)	Histopathology	Genotyping array	Ethnicity	% Female (cases/controls)	Median Age (cases/controls)
Discovery						
UCSF Adult Glioma Study	620 / 602	Glioblastoma (85%)[a]	Illumina 370k	Caucasian	36% / 47%	56 / 57
TCGA glioblastoma cases	70 / 0	Glioblastoma	Illumina 550k	Caucasian	43% / -	55 / -
Illumina iControls	0 / 3390	-	Illumina 370k/550k	Caucasian	- / 63%	- / 31
Validation						
TCGA glioblastoma cases	323 / 0	Glioblastoma	Affymetrix 6.0	Caucasian	38% / -	60 / -
TCGA lower-grade glioma	176 / 0	Grade II/III Glioma[b]	Affymetrix 6.0	Caucasian	41% / -	43 / -
Wellcome Trust controls	0 / 2603	-	Affymetrix 6.0	Caucasian	- / 48%	- / -[c]

[a] 15% of the high-grade glioma samples were Grade 3 anaplastic astrocytomas

[b] 35% astrocytoma, 39% oligodendroglioma, 25% mixed oligoastrocytoma

[c] Precise age data were unavailable, but >50% of the included samples were members of the 1958 UK Birth Cohort.
